# Old World Monkeys Compare to Apes in the Primate Cognition Test Battery

**DOI:** 10.1371/journal.pone.0032024

**Published:** 2012-04-02

**Authors:** Vanessa Schmitt, Birte Pankau, Julia Fischer

**Affiliations:** 1 Cognitive Ethology Lab, German Primate Center, Göttingen, Germany; 2 Courant Research Centre Evolution of Social Behaviour, University of Göttingen, Göttingen, Germany; University of Florence, Italy

## Abstract

Understanding the evolution of intelligence rests on comparative analyses of brain sizes as well as the assessment of cognitive skills of different species in relation to potential selective pressures such as environmental conditions and social organization. Because of the strong interest in human cognition, much previous work has focused on the comparison of the cognitive skills of human toddlers to those of our closest living relatives, i.e. apes. Such analyses revealed that apes and children have relatively similar competencies in the physical domain, while human children excel in the socio-cognitive domain; in particular in terms of attention sharing, cooperation, and mental state attribution. To develop a full understanding of the evolutionary dynamics of primate intelligence, however, comparative data for monkeys are needed. We tested 18 Old World monkeys (long-tailed macaques and olive baboons) in the so-called Primate Cognition Test Battery (PCTB) (Herrmann et al. 2007, Science). Surprisingly, our tests revealed largely comparable results between Old World monkeys and the Great apes. Single comparisons showed that chimpanzees performed only better than the macaques in experiments on spatial understanding and tool use, but in none of the socio-cognitive tasks. These results question the clear-cut relationship between cognitive performance and brain size and – prima facie – support the view of an accelerated evolution of social intelligence in humans. One limitation, however, is that the initial experiments were devised to tap into human specific skills in the first place, thus potentially underestimating both true nonhuman primate competencies as well as species differences.

## Introduction

Understanding the evolution of human cognition and communication rests primarily on comparative analyses with other extant members of the primate order. There are two major and interrelated streams of research; one focuses on the evolution of the brain, while the other aims at elucidating similarities and differences in behaviour. Such analyses thus incorporate information about the phylogenetic relationships between species as well as the putative selective pressures that might have played a role in shaping a species' cognitive skills. Within the hominoidea (apes and humans), the last common ancestor of humans and their closest relatives, the chimpanzees and bonobos, is dated at about 6 mya [1], while the split between the Hominoidea and the Cercopithecoidea (Old World monkeys) occurred between 29 and 24 mya [2]. Taking this phylogenetic information into account is a prerequisite for identifying the dynamics in the evolution of specific adaptations. One striking feature within the primate order is a disproportionate increase in relative brain size from monkeys to apes to humans [3]. In particular, the neocortex has experienced considerable expansion. The neocortex is important for sensory perception, generation of motor commands, and higher cognition [4]. In the 1980s, the most prominent hypothesis was that the increase in brain size in primates was related to frugivory, that is, the need to find food that is patchily distributed in space and time [5]. In recent years, the focus has returned to the idea that primate intelligence evolved in response to the challenges of living in large and complex groups – the so-called “Social Brain” hypothesis [6]–[12].

Whether this increase in brain size at the same time predicts an increase in cognitive abilities remains controversial. For instance, it has been proposed that overall brain size best predicts the cognitive abilities across nonhuman primates [13], [14]. More recently, a number of scholars have aimed to derive more specific links between particular brain areas and cognitive performance. Shultz and Dunbar [15], for example, claimed that the neocortex ratio and hippocampus volume are particularly important for problem solving and executive control. Others, however, have pointed out that attempts to link brain size to function is fraught with problems, including the choice of the variables entered in the analyses, and the problems associated with multiple correlations [16]. Further, size per se might not be the critical factor, but in fact the modularity and interconnectedness of different brain areas [17].

Yet, it is undisputed that human brains are disproportionately larger than the brains of other primate species. In line with this, a systematic comparison of the cognitive skills of human toddlers and great apes revealed substantial differences in cognitive performance [18]. Subjects were tested in largely identical experiments (the so-called Primate Cognition Test Battery [PCTB]). While great apes and children showed relatively similar competencies in the physical domain (space, quantities, causality), human children excelled in the socio-cognitive tasks; in particular in terms of attention sharing, cooperation, and mental state attribution. This supports the assumption that social aspects were the driving force in the evolution of intelligence, at least in the transition from apes to humans.

To develop a full understanding of the evolutionary dynamics of primate intelligence, however, comparative data for monkeys are needed [19]. With the increase in brain size from monkeys to apes one would predict that apes would outperform monkeys in cognitive tasks. Indeed, Byrne and Whiten [6] noted for example that tactical deception seems to be more common in great apes than in monkeys. Furthermore, only great apes recognize themselves in mirrors [20], [21], lending further support for the distinction between monkeys and apes. A meta-analysis of published nonhuman primate cognition studies also indicated that “great apes significantly outperformed other lineages” (p. 115) in their overall performance [22].

In contrast to these results, a recent study by Amici and colleagues [23] suggested that the cognitive abilities of monkeys and apes are not so different. They compared the performance of three monkey species (spider monkeys, capuchin monkeys, long-tailed macaques) and all four great ape species in spatial displacement and support tasks (i.e. using for example an unbroken cloth to pull in a reward) and found no support for a clear-cut difference between apes and monkeys. Notably, an additional analysis focusing on inhibition tasks revealed that species living in systems with fission-fusion dynamics (chimpanzees, bonobos, orangutans, and spider monkeys) outperformed members of species that live in more stable groups (long-tailed macaques, gorillas and capuchin monkeys). Apparently, the level of social complexity predicted the inhibitory skills better than phylogenetic relatedness or ecological conditions [24]. One possible explanation for the discrepant assessments of the differences between monkeys and apes may be that the (meta-) analyses incorporated results of experiments or observations made in different studies using different methods. Furthermore, the differences between monkeys and apes may have been overestimated, because in many studies highly trained apes were compared to naive monkeys [25]. Thus, although more comparative studies are now available [26], systematic interspecific comparisons are still rare.

The differences in results may also be due to the fact that different tests may tap into different cognitive domains. In other words, there may be no increase in general intelligence from monkeys to apes, but more domain specific differences. Interestingly, Amici and colleagues [23] found no clear distinction between monkeys and apes in their spatial memory, transposition, and support tasks, but what remains unknown is whether there are differences between the two lineages regarding other cognitive aspects. For instance, in the experiments by Herrmann et al. [18] great apes and children did not differ in their physico-cognitive capacities, but only in the experiments relying on social cognition. Perhaps this is also the case in the transition from monkeys to apes. Thus, we set out to systematically compare the skills of monkeys to that of apes, applying the same test as Hermann and colleagues on apes and toddlers. We therefore conducted the complete suite of experiments of the Primate Cognition Test Battery with Old World monkeys (olive baboons and long-tailed macaques) housed at the German Primate Center and compared them to the results of great apes. The data for the apes were kindly made available to us by Hermann and colleagues.

If an increase in brain size predicts an overall increase in cognitive performance, we would hypothesize that the monkeys perform less well than the apes in all experiments. In contrast, if an increase in brain size is (more or less) linearly related to an increase in socio-cognitive skills, then we would predict that the apes outcompete the monkeys especially in the socio-cognitive tasks, while they should perform on a more or less comparable level in the physical domain. However, it might also be the case that the human lineage underwent a nonlinear increase in socio-cognitive skills, in which case we would predict that apes and monkeys do not reveal substantial differences in either of the cognitive domains. As recent studies have shown further factors can influence the performance in cognitive tasks such as a shy or bold temperament [27], [28] or the amount of inhibitory control [24], [29]. To control for those aspects we included the temperament and inhibitory control experiments of Herrmann et al. [18] in which we measured the subject's reaction to novel objects, people, and rewards, and their ability to control their impulses in a spatial memory task. In relation to the previous studies we expected to find an influence of these parameters on the cognitive performances of the monkeys.

## Materials and Methods

### Ethics Statement

All testing was non-invasive and the subjects participated voluntarily in the experiments. They were not food deprived for testing and water was always available ad libitum. All experiments were performed under the control of experienced veterinarians to ensure that the studies were in accordance with the NRC Guide for the Care and Use of Laboratory Animals and the European Directive 2010/63/EU on the protection of animals used for scientific purposes. Furthermore, in accordance with the German Animal Welfare Act and corresponding section for animals used for scientific purposes, the study approval was checked by the responsible Animal Welfare Officer of the German Primate Center (Permit Number 33.9-42502).

### Subjects

We tested 13 long-tailed macaques (*Macaca fascicularis*) - 6 males and 7 females aged 1 to 7 years (M_age_ = 2.8 years) - living in a social group of 28 animals and 5 olive baboons (*Papio anubis*) -3 males and 2 females aged 3 to 9 years (M_age_ = 6.1 years) - living in a social group of 11 animals. The monkeys were housed at the German Primate Center in Göttingen and had access to indoor (baboons: 17 sqm, macaques: 40 sqm) and outdoor areas (baboons: 81 sqm, macaques: 141 sqm). The subjects were individually tested in their familiar indoor cages. Before the testing began all animals were trained to be separated from the group using positive reinforcement. One session lasted about 10 to 15 minutes. If an animal was not willing to participate in a session (e.g. not choosing a reward option) it was released again to the group and tested on another day.

### Primate Cognition Test Battery

As the aim of this study was to conduct a systematic interspecific comparison, we used the same experimental procedures of the so-called Primate Cognition Test Battery (PCTB) as Herrmann and colleagues [18]. The PCTB consists of 16 tasks examining skills of physical cognition, i.e. an understanding of objects and their spatial, numeral and causal relationships, as well as of social cognition, i.e. an understanding of other animate beings and their intentional actions, perceptions, and knowledge. The 16 tasks are grouped into six scales. Three of these scales belong to the physical domain: Space, Quantity, and Causality. In these experiments we tested the monkeys' understanding of spatial displacements, their quantity discrimination abilities, and their understanding of the causal relations between two objects. The other three scales belong to the social domain: Social learning, Communication, and Theory of Mind. In these experiments we examined whether the monkeys imitate simple tasks such as shaking a reward out of a tube, understand communicative cues and intentional actions, as well as whether they follow the gaze of a human.

In contrast to Herrmann et al. we applied control conditions to some of the tasks and new quantity combinations in the quantity discrimination experiments (see File S1 for a detailed description of the methods). We adjusted the size of the material used to be operable for the baboons and long-tailed macaques, respectively. To facilitate the comparison of our results with those of Herrmann and colleagues, we here applied the same terminology as in the previous study. In the discussion, we will critically evaluate some of the connotations associated with the terms used for these experiments.

In the following we will shortly outline the experimental procedure of the 16 tasks of the PCTB. Some tasks consist of different items, which are described in detail in the Supporting Information and Herrmann et al [18].

### Physical Domain

#### Space

To test the monkeys' ability to track specific objects while they were being displaced in various ways, we conducted different ‘spatial displacement’ tasks. In total this scale is made up of four different tasks: *Spatial Memory*, *Object Permanence*, *Rotation*, and *Transposition*. In each task, three cups were aligned in a row on the testing tray and manipulated differently: To test their *Spatial Memory* two rewards were placed under two of the three cups and the subject was allowed to choose twice. In the *Object Permanence* task a small opaque cup, which contained a reward, was moved under one or two of the three larger cups in succession, leaving the reward under one of these at the end. The subject had to track these operations to locate the reward. We conducted an additional control condition in which the experimenter also touched the cups under which the smaller cup was not moved with her hand to examine whether the subjects only chose the last cup touched by the experimenter or really took into account where the smaller cup was moved to. In the *Rotation* task three cups, one containing the reward, were aligned on a moveable tray, which than was rotated 180° and 360°. The subjects had to follow the rotation to locate the reward. In the *Transposition* task the position of the baited cup was switched with the position of the other cups in three different ways. The subjects had to follow these transpositions to locate the reward.

#### Quantities

To test the monkeys' abilities to discriminate between different food amounts, we conducted different two-choice experiments where they received the amount of food pieces they had pointed at. This scale consisted of two tasks: *Relative Numbers* and *Addition Numbers*. In the so-called *Relative Numbers* task the monkeys could choose between 1 and 8 food pieces lying on two different plates with differences between the two amounts ranging from 1 to 4 pieces. In the so-called *Addition Numbers* task the subjects were shown three different amounts of food items. The food items from the center plate were transferred to one of the side plates after a few seconds. The subjects had to choose the resulting larger number to be scored as a correct response.

#### Causality

To test their understanding of the spatial-causal relationships between two objects the monkeys were tested in four different tasks: *Noise*, *Shape*, *Tool Use*, and *Tool Properties*. In the *Noise* task, the subjects had to choose one of two cups. To give them a hint where the reward was located the cups were shaken. One cup contained a peanut and made a rattling sound when shaken. In the *Shape* task either two plastic boards or two pieces of cloth were placed on the tray. A reward was placed under one of the boards or cloths causing a visible bump, and the subjects were allowed to choose. To test their *Tool Use* abilities a reward was placed on the tray out of reach of the subject and a wooden stick was provided to the subject. The subject had to use the tool to retrieve the out of reach food. In the *Tool Properties* task a functional and a non-functional tool were presented. For example, a reward was placed on top of one piece of cloth, whereas the other reward was placed directly next to the other cloth piece. The subjects were allowed to pull one of the two pieces. Altogether, five different items were used in this task (cloth: food was placed on top or right next to a piece of cloth; Plexiglas bridge: a small bridge was placed over a piece of cloth; food was placed on top of the bridge or underneath directly onto the cloth; ripped cloth: food was placed on an intact or a ripped piece of cloth; broken wool: food was tied to the end of an intact or cut string of wool; tray circle: food was placed into a cardboard piece with a round hole in it or with a u-shaped opening, an attached string allowed the monkeys to pull the tray).

### Social Domain

#### Social Learning

To test whether the monkeys' imitate simple actions done by a human to get food three different items were used. In all experiments a human demonstrator showed the subjects how to open three different plastic tubes which contained a reward (*Paper tube, Banana tube, Stick tube*). We scored whether the subjects solved the problem by the same means as the demonstrator. The behaviour of the subjects was compared to that of a control group (3 baboons and 3 macaques) who were given the opportunity to open the tubes without prior exposure to a human demonstrator (baseline condition).

#### Communication

To test their ability to use communicative cues by humans, the subjects were tested in three different tasks: *Comprehension, Pointing Cups*, and *Attentional State*.

The *Comprehension* task consisted of a two-choice paradigm in which the experimenter gave different cues to locate the reward. She either looked or pointed at the cup, which contained the reward or – in the control condition - placed an iconic marker (e.g. picture of a peanut) on it. The animal was then allowed to make its choice.

In the two tasks under the umbrella term *Production* two experimenters were needed. In the *Pointing Cups* task, one experimenter baited one of two cups, which were placed about 70 cm apart and left the room. Then the second experimenter entered the room. We then scored whether the subject indicated its choice by pointing at a cup. In the *Attentional State* task the attentional state of the main experimenter varied in four different ways. A second experimenter first placed a reward in front of the subject's cage and left the room. When the main experimenter entered the testing area, she either turned around and looked away from the reward, looked towards the reward, turned towards the reward but looked away or turned away from the reward but looked at it. The subject had to draw the experimenter's attention to the reward (e.g. by moving into her visual field and reaching for the reward) in order to receive it.

#### Theory of Mind

The experiments under this umbrella term encompassed experiments in two different tasks: *Gaze Following* and *Intentions*. In the *Gaze Following* task the experimenter sat in front of the monkey, hid a piece of food in her hands, and then completed three different actions: She held her hands in front of her body and looked up with her head and eyes; she sat with her back facing the subject, holding her hands next to her shoulders and looked up to the ceiling; or she held her hands in front of her body and glanced with her eyes only up to the ceiling. A response was scored if the subject followed the gaze of the experimenter and looked up. The behaviour of the subjects was compared to a baseline condition in which we measured how often the monkeys gazed upwards when the experimenter looked straight at the subject's chest (we did not stare directly at their eyes as this is a threatening behaviour in monkeys).

In the *Intentions* task, the experimenter tried to retrieve a reward out of one of two cups but failed. In the first test, she tried in vain to remove a lid; in the second test, a Plexiglas barrier blocked her access to the cup. The subject was then allowed to choose one of the cups.

### Testing Apparatus and General Procedure

To test the cognitive capacities of the animals, they were separated from their group in their familiar indoor compartments. The testing apparatus used in most of the experiments (when other material was used it is indicated in the description of the experiments in the Supporting Information) consisted of a sliding board made of grey polyvinylchloride (length 0.8 m, width 0.27 m, height 0.01 m (baboons); length 0.55 m, width 0.2 m, height 0.01 m (macaques)), which was attached to a fixed polyvinylchloride table (length 0.8 m, width 0.38 m, height 0.01 m (baboons); length 0.55 m, width 0.3 m, height 0.01 m (macaques)) by two drawer rails so that the sliding table could be moved horizontally. Three white opaque cups (Ø 7.5 cm×7.5 cm height) or other materials (which are reported in File S1) were used to cover/present the food reward. These were placed on the sliding table. The sliding table was attached with an iron mount in front of a plastic panel. The middle of the plastic panel was cut out, which allowed one of two different kinds of plastic slices to be inserted, depending on the tasks performed. One of the plastic slices had three holes in it (Ø 1 cm, distance 20 cm (baboons), distance 15 cm (macaques)) that allowed the subjects to point with their fingers at the cups. The other slice had two oval openings at the outer sites (5.5 cm×2.5, distance 25 cm (baboons), 5.5 cm×1.5 cm, distance 30 cm (macaques)) to allow the subjects to retrieve e.g. pieces of cloth. Depending on the tasks, one of the two slices was attached to the panel.

Throughout testing, unless otherwise indicated, a choice was scored when the subjects pointed with one finger at one of the locations or put their fingers through one of the oval openings to retrieve e.g. cloth after the sliding table had been pushed against the Plexiglas panel. When the monkeys indicated the correct location, they were given a small food reward. However, unless otherwise stated, when they made incorrect responses they were always shown the location of the hidden food after each trial. The same desirable food items were used as rewards for most of the tasks (raisins, peanuts, pieces of fruits). It was possible to set up an occluder of grey plastic (length 0.8 m, height 0.3 cm, thickness 0.03 m) in front of the panel so that the subjects were not able to watch the baiting procedures. All sessions were videotaped with a digital video camera (Sony DCR-HC90E). A naïve second observer coded 20% of all videotapes to assess inter-observer reliability, which was excellent (Cohen's K = .97, N = 809).

### Design

In general, we followed the design of Herrmann et al. [18] but doubled the number of trials in the object-choice tasks (see File S1) in order to include all possible spatial positions and combination of locations to control for the influence of using only a subset of all possible manipulations (see description of the specific tasks for details). Furthermore, we wanted to reduce the risk of obtaining significant effects by chance due to our smaller sample size in combination with a very small number of trials. We also controlled for learning over the trials, but did not find any effects in any of the tasks (Pearson Correlations between Trial and Performance; note however that only a small number of trials were conducted per condition and individual). For the other tasks, i.e. those that did not include object choice (Social learning, Attentional State and Gaze following), we applied the same number of trials as in the study by Herrmann and colleagues (2007) since in these experiments, the subjects had to perform specific actions, which probably did not happen coincidentally (see also control experiments for Social learning and Gaze following).

Furthermore, we pseudo-randomized and balanced the order of administering the experiments of the different scales across individuals to exclude any order effect. Eight of the individuals started with the experiments of the physical domain (5 baboons and 3 macaques) and ten started with the tasks of the social domain. There was no difference in the performance of the monkeys in relation to whether they started with the social or physical domain (ANOVA with first domain as between subject factor and performance in the two domains as dependent variables, F (2, 15) = 0.28 p = .757, η^2^ = .036). Within each scale and task, the order of the experiments was also pseudo-randomized and balanced across individuals.

All macaques were completely naive to cognitive testing and working with a human experimenter prior to these experiments. The baboons had already participated in an experiment on size discrimination (manuscript in preparation) and a study about inferential reasoning [30].

### Influence of Temperament, Inhibitory Control and Rank on Performance

To test whether differing temperaments, inhibitory control or rank positions influenced the monkeys' performances in the test battery, each subject participated in a set of additional tests comparable to those used by Herrmann et al. [18] (see File S2 for detailed descriptions). In terms of temperament we measured the subjects approaching behaviour to novel objects, people and foods. Their amount of inhibitory control was examined during an additional spatial memory task, which specifically assessed whether the monkeys are able to skip the middle out of three cups. To assess the influence of rank we classified each individual as high, middle or low ranking on the basis of focal observations done by V.S..We then tested whether the results of these measurements correlated with the performance of the monkeys in the PCTB (Pearson correlations).

### Data analysis

First, we determined the overall proportion of correct responses in each task for every subject. To measure whether the baboons and macaques performed above chance level or baseline we used the Wilcoxon-test because of the small sample sizes (to correct for multiple testing we applied a Benjamini-Hochberg correction). On the individual level we used Binomial-tests to see whether the performance was significantly better than expected by chance. To explore whether there were significant differences between the performances of the baboons and macaques in the test battery we conducted multivariate analysis of variance (MANOVA) with species and sex or rank as between-subject factor and performance of the baboons and macaques in each task as dependent variable. In case of significant effects we controlled for age by using analysis of covariance (ANCOVA). To further compare the performance of the baboons and macaques in each task we used univariate analysis of Variance (ANOVA) or in cases when data were not normally distributed (normality test using Shapiro Wilks-tests) a Kruskal-Wallis ANOVA. In case of significant effects, post-hoc tests (Bonferroni) were conducted. For the tool use task no statistical analyses were possible as performance was zero for all subjects. As we did not have information about the performance of the apes in each task, we only conducted repeated measures ANOVAs on the scale level, with follow-up post-hoc tests (Bonferroni) to compare the performance of the four species (baboon, macaque, chimpanzee, orangutan). The critical p-value was set to α = .05 (except for pair wise comparisons) and all tests were two-tailed.

## Results

### Performance in the different Tasks

#### Space

**Figure 1:**
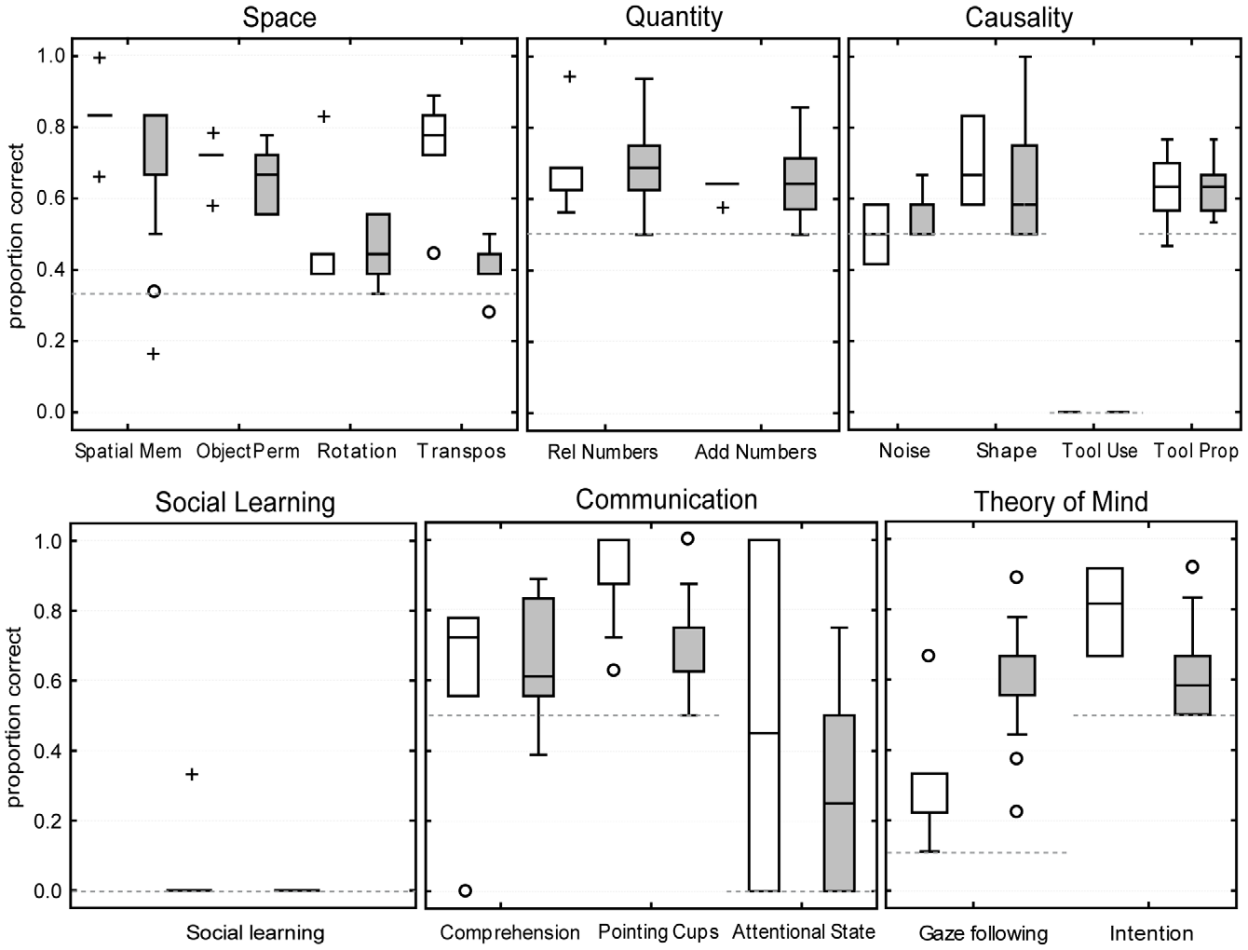
Performance of the monkeys in the PCTB. Shown are the proportions of correct responses of the baboons (white) and macaques (grey) in the 16 tasks of the PCTB grouped into the respective scale. Boxes show the interquartile range from the 25^th^ to the 75^th^ percentile. The line across the boxes represents the median. The whiskers indicate the maximum and minimum values excluding outliers (circles) and extreme values (crosses). The dotted lines represent the chance level and baseline, respectively for each task.

On the species level both baboons and macaques performed significantly above chance level (0.33) in all four tasks of the scale Space (see Figure 1) (macaques: Spatial Memory (*z* = 3.04, adjusted *p* = .012), Object Permanence (*z* = 3.18, adjusted *p* = .022), Rotation (*z* = 3.18 adjusted *p* = .015); and Transposition (*z* = 2.76, adjusted *p* = .016); baboons: Spatial Memory, Object Permanence, Rotation and Transposition (all *zs* = 2.02, all adjusted *ps* = .049)). The macaques also performed above chance level in the control task we conducted during the Object Permanence tests (*z* = 2.93, adjusted *p* = .012), and the performance of the baboons also nearly reached significance (*z* = 1.83, adjusted *p* = .075). On the individual level, however, none of the macaques performed above chance in the transposition task, whereas four out of five baboons did (see Table 1).

**Table 1:** Mean proportion of correct responses of the baboons and macaques in each task (*and Scale*) of the PCTB.

		macaques				baboons				
Tasks	Trials	n	M (SD)	95% CI	Ind	n	M (SD)	95% CI	Ind	Chance
***Space***			.*54 (.06)*	*[.51,.58]*			.*69 (.03)*	*[.65, .73]*		
Spatial Mem	6	13	**.68** (.22)	[.55, .81]	7	5	.**83** (.12)	[.69, .98]	4	.33
Object Perm	18	13	**.65** (.08)	[.60, .70]	13	5	**.71** (.06)	[.64, .79]	5	.33
Rotation	18	13	**.46** (.09)	[.41, .51]	5	5	**.49** (.19)	[.25, .73]	1	.33
Transposition	18	13	**.39** (.07)	[.35, .44]	0	5	.**73** (.17)	[.52, .95]	4	.33
***Quantity***			.*67 (.08)*	*[.62, .72]*			.*66 (.06)*	*[.59, .73]*		
Rel Numbers	16	13	**.70** (.13)	[.62, .78]	4	5	**.69** (.15)	[.51, .87]	1	.50
Add Numbers	14	12	**.64** (.11)	[.57, .71]	2	5	**.63** (.03)	[.59, .67]	0	.50
***Causality***			.*46 (.05)*	*[.43, .50]*			.*46 (.04)*	*[.41, .51]*		
Noise	12	13	.56 (.07)	[.51, .60]	0	5	.50 (.08)	[.40, .60]	0	.50
Shape	12	13	**.66** (.17)	[.56, .76]	2	5	**.70** (.13)	[.54, .86]	2	.50
Tool Use	1	13	0		0	5	0		0	.00
Tool Prop	30	13	**.64** (.08)	[.59, .68]	4	5	.63 (.12)	[.48, .77]	2	.50
***Social learning***	*3*	*10*	*0*		*0*	*5*	.*07 (.15)*	*[−.12, .25]*	*0*	.*00* a
***Communication***			.*53 (.13)*	*[.45, .61]*			.*69 (.21)*	*[.42, .95]*		
Comprehension	18	13	**.66** (.17)	[.56, .76]	4	5	**.71**(.09)	[.60, .82]	4	.50
Pointing Cups	8	13	**.69** (.15)	[.60, .78]	3	5	**.90** (.16)	[.70, 1.10]	4	.50
Attention State	4	13	**.23** (.26)	[.07, .39]	0	5	.45 (.51)	[−.19, 1.09]	2	.00
***Theory of Mind***			.*59 (.10)*	*[.53, .65]*			.*57 (.13)*	*[.41, .74]*		
Gaze following	9	13	**.57** (.17)	[.47, .67]	12	5	**.33** (.21)	[.08, .60]	3	.10^a^
Intention	12	13	**.62** (.14)	[.53, .70]	2	5	**.82** (.11)	[.68, .95]	3	.50

Note: Significant deviations from chance level are in boldface (α = .05). Performance on the scale level was not compared to chance as this varies between tasks. **Trials** = Number of trials performed in each task; **n** = Number of tested individuals; **Ind** = Number of individuals performing above chance level; **CI** = confidence interval;

a results of the baseline conditions.

#### Quantity

Both species performed significantly above chance level (0.5) in the two tasks on quantity discrimination (see Figure 1) (macaques: Relative Numbers (*z* = 3.06, adjusted *p* = .013), Addition Numbers (*z* = 2.80, adjusted *p* = .017); baboons: Relative Numbers (*z* = 2.02, adjusted *p* = .049), Addition Numbers (*z* = 2.02, adjusted *p* = .049)). On the individual level none of the baboons, but two macaques performed above chance in the Addition Number task (see Table 1).

#### Causality

None of the baboons or macaques solved the tool use task where they had to use a stick to retrieve food. However, on the species level the macaques performed above chance (0.5) in two other tasks of the scale Causality: Shape (*z* = 2.67, adjusted *p* = .019), and Tool properties (*z* = 3.18, adjusted *p* = .044) and nearly reached significant values in the task Noise (*z* = 2.20, adjusted *p* = .055) (see Figure 1). The baboons however only performed significantly above chance in the Shape condition (*z* = 2.02, adjusted *p* = .049).

#### Social learning

In the baseline condition, where the subjects did not get any demonstration on how to open the different tubes, none of the six subjects used a method similar to the one demonstrated in the test condition. In this condition, neither the baboons nor the macaques showed any evidence of social learning. Only once did one baboon use a similar technique as the human demonstrator, but that does not deviate significantly from the baseline (0.0) (see Figure 1).

#### Communication

The macaques performed significantly above chance level in all three tasks of the scale Communication, i.e. Comprehension (chance 0.5, *z* = 2.83, adjusted *p* = .014), Pointing Cups (chance 0.5, *z* = 2.93, adjusted *p* = .012), and Attentional State (chance 0, *z* = 2.37, adjusted *p* = .038). The baboons performed significantly above chance only in the Comprehension (*z* = 2.02, adjusted *p* = .049) and Pointing Cups tasks (*z* = 2.02, adjusted *p* = .049), but not in the Attentional State condition (*z* = 1.60, adjusted *p* = .113) (see Figure 1). However, none of the baboons performed above chance in the Gaze and Point condition of the Comprehension task, but three macaques did (Binomial-tests, *p* = .016). In contrast, none of the macaques performed significantly above chance in the Attentional state task (see Table 1), whereas two of the baboons scored a 100% correct.

#### Theory of Mind

Considering gaze following both baboons and macaques performed significantly above baseline, which we assessed by the monkeys' looks upwards while the experimenter was looking straight (they looked upwards in M = 10 percent of all trials). In the test situation the macaques followed the human gaze very often (M = .57, *z* = 3.18, adjusted *p* = .011), whereas the baboons did so a bit less (M = .33, *z* = 2.02, adjusted *p* = .049) (see Figure 1). Both species also performed significantly above chance level (0.5) in the task on understanding intentions (macaques: *z* = 2.52, adjusted *p* = .027; baboons: *z* = 2.02, adjusted *p* = .049). On the individual level 12 macaques and three baboons followed the human gaze significantly more often than in the baseline condition, whereas only two out of 13 macaques performed above chance in the Intention task, but three out of five baboons did (see Table 1).

### Comparison of Baboons and Macaques

As none of the baboons or macaques solved the tool use task, we had to exclude it from the following statistical analysis of variance. Considering the performance in the other 15 tasks of the PCTB a multivariate analysis of variance revealed no significant differences between the two species (MANOVA with species and sex as between-subject factor and performance in the 15 different tasks as dependent variables; Wilk's Lambda, *F* (11,1) = 4.88, *p* = .346, η^2^ = .982). However, as Figure 1 indicates, there was a large difference between the species in the transposition task, and univariate analyses indeed revealed that here the baboons performed significantly better than the macaques (Kruskal-Wallis ANOVA, *H* (1, N = 18) = 8.10, *p* = .004) also when age was controlled for (*F* (1, 15) = 119.61, *p*<.001, η^2^ = .889). There were no significant differences between the species in any other tasks after correction for multiple testing (all ps>.01).

### Influence of Temperament, Inhibitory Control and Rank

We found no significant correlations between the temperament measures and the performance of the monkeys in the two domains for either macaques (social domain (*r* (12) = −.27, *p* = .395), physical domain (*r* (12) = .18, *p* = .576); nor baboons (social domain (*r* (5) = .66, *p* = .229), physical domain (*r* (5) = .58, *p* = .299). Furthermore, there were no significant correlations between the performance in the social and physical domain and the inhibitory control task for the macaques (social domain: Spearman correlations (13) = −.18, *p* = .565; physical domain: Spearman correlations (13) = .04, *p* = .892) and baboons (social domain: Spearman correlations (5) = .58, *p* = .308; physical domain: Spearman correlations (5) = .00, *p* = 1). Rank and sex had also no effect on the performance of the monkeys in the PCTB (rank: *F* (18, 2) = 1.84, *p* = .409, η^2^ = .943; sex: *F* (11, 1) = 2.15, *p* = .491, η^2^ = .959).

### Comparison of Monkeys and Apes

**Figure 2:**
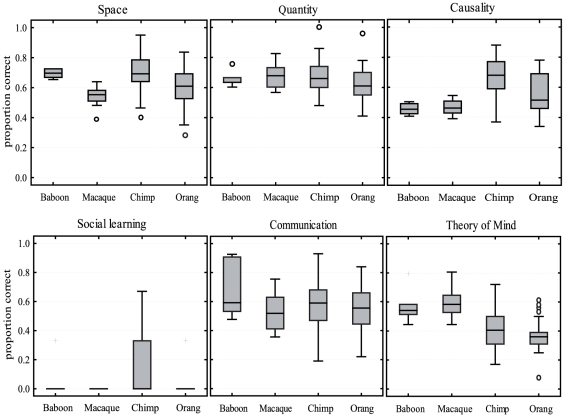
Comparison of species. Shown are the proportions of correct responses on the scale level for the four different primate species. Boxes show the interquartile range from the 25^th^ to the 75^th^ percentile. The line across the boxes represents the median. The whiskers indicate the maximum and minimum values excluding outliers (circles) and extreme values (crosses).

To compare the performances of the four species (baboons, macaques, chimpanzees, orangutans) we calculated the mean proportion of correct responses in each scale for the two monkey species and compared this to the results for the chimpanzees and orangutans taken from Herrmann et al. [18] (Figure 2). Statistical analysis revealed a significant effect of species (repeated measures ANOVA; Wilk' Lambda, *F* (18, 407.78) = 6.09, *p*<.001, η^2^ = .211). Post-hoc test (Bonferroni), however, showed that there were no significant differences between monkeys and apes in the scales Quantity, Social learning and Communication (all *ps>.266*). The chimpanzees performed significantly better than the macaques only in the scales Space and Causality (Post-hoc tests, space *p*<.001, causality *p*<.001) and better than the baboons only in the scale Causality (Post-hoc test, *p* = .005). However, the differences in the scale Causality were mainly due to the ‘tool use’ task, which none of the monkeys solved. Looking at the scale Causality without including the tool use task there were no significant differences between the species (Posthoc tests, *p* = 1). In the scale Theory of Mind the macaques performed significantly better than the chimpanzees (*p*<.001) and orangutans (*p*<.001), and the baboons performed significantly better than the orangutans (*p* = .002). There were no significant differences between the baboons and chimpanzees after correction for multiple testing in this scale.

## Discussion

The results of our experiments indicate that olive baboons and long-tailed macaques have a very good understanding of objects and their spatial, numeral, and causal relations. Both monkey species performed above chance in all tasks on spatial displacements and quantity discrimination and showed only some limitations in experiments on causal understanding (e.g. tool use). An analysis of the performance in the social domain reveals a more inconsistent picture. Although the monkeys followed the gaze of the human experimenter significantly more often than in the baseline condition, they only marginally used the gazing cue in the object-choice task. Furthermore, members of both species did not show any indication of imitation in the social learning tests and only two baboons seemed to show some understanding of the attentional state of the experimenter. In contrast the monkeys performed very well when intentional actions of the experimenter served as a cue in an object choice task. However, as this can also be explained by simply using local enhancement, this result should be interpreted with caution (see below).

The good performances of our monkeys in the physical domain are in line with a recent comparative study on New and Old World monkeys, which found no clear-cut distinction between the capacities of monkeys and apes [23]. Comparing the performance of our monkeys to that of great apes in the physical tasks of the PCTB also revealed no distinct differences between the two taxa (i.e. Hominoidea and Cercopithecoidea). However, we found cognitive differences between particular species, especially in tasks on spatial transpositions. The olive baboons in our experiments outperformed all three monkey species (spider monkeys, capuchin monkeys, long-tailed macaques) tested by Amici and colleagues [23], mirroring our finding that the baboons performed significantly better than the macaques in the Transposition task. This is particularly interesting as mastering these fairly demanding object displacement tasks has not yet been reliably shown in Old World monkeys and the ability has long been used as the main type of data to support the distinction between apes and monkeys [22]. We, however, found no significant differences between the performances of baboons and great apes in these tasks. Interestingly Herrmann et al. [18] also found significant differences in the scale Space between chimpanzees and orangutans. Thus, although there are differences between species in this aspect, there seems to be no deep split between apes and monkeys. Recently, differences in cognitive capacity have been linked to social organisation. Specifically, it was suggested that subjects living in fission-fusion societies may exhibit enhanced cognitive skills [23], [24]. Constant fission and fusion among subgroups is thought to require enhanced memory, inhibitory control and analogical reasoning as subjects are permanently confronted with changing group compositions [31]. For our two species, however, this explanation does not apply as both olive baboons and long-tailed macaques live in stable female-bonded groups. Why certain species do better than others in spatial tasks may also have something to do with the foraging techniques used, but this issue requires further empirical investigation.

Furthermore, we found no differences between monkeys and apes in the quantity discrimination tasks, and the differences in the scale Causality were mainly due to the monkeys failing to use a stick to retrieve out-of-reach food. In contrast to the finding by Amici and colleagues [23], however, our long-tailed macaques performed reasonably well in the tasks on tool properties. Indeed, long-tailed macaques have been reported to use tools in the wild, supporting the assumption that they should have some understanding of the causal relations between an object and food. For instance, they use stones to crack open oysters or crabs and do so quite efficiently [32], [33]. That they failed in the Tool Use task of the PCTB may therefore be due to the high difficulty of this task as it requires quite fine scaled motor control and may have had too little ecological relevance for the monkeys. These results further support the view that the physico-cognitive capacities of monkeys and apes are not that distinct in general, but that differences between species exist in more specific aspects, which may be better explained by socio-ecological aspects than by phylogenetic relationships [23].

Concerning the tasks of the social domain, we also did not find an increase in performance from monkeys to apes. The long-tailed macaques even scored significantly higher than the apes in the Theory of Mind scale. However, despite the fact that the monkeys did well in the Theory of Mind tasks, it should be noted that most of the tasks can be solved without attribution of mental states. For instance, gaze following can be conceived as a simple orienting reflex or somewhat more elaborate as behaviour reading (for a study on gaze following see e.g. [34]). Thus, the extensive gaze-following behaviour of the macaques does not imply an enhanced understanding of other minds, especially in comparison to the baboons and apes. The macaques seemed to be more re-active and tuned to the experimenter during the gaze following experiments, probably leading to a slowed habituation.

The Pointing Cups and Intention tasks also consisted of two-choice tests, which could have been solved by just behaviour reading or by using spatial associations as e.g. the proximity between the experimenter's hand and the cup (for a discussion on behaviour reading see e.g. [35], [36]). Furthermore, the baboons had already participated in two-choice experiments before and it may well be that their former experience with human subjects in this kind of setup improved their performance. To summarize, we do not imply that our subjects attribute mental states to others, and only chose this terminology to remain consistent with previous studies [18].

Taken together, our experiments neither showed an increase in general intelligence nor in socio-cognitive abilities from Old World monkeys to apes, contradicting the theory that an increase in brain size is necessarily linked to an increase in intelligence [22]. In contrast, the species differences we found were on a more domain specific level (e.g. spatial displacements) with variation between but also within taxa. These findings may be somewhat surprising as a number of studies claimed that there is a large difference between apes and monkeys, in particular in their ability to form mental representations [37], i.e. to hold in mind and operate on mental objects that have semantic properties [38]. Whereas the cognitive system of great apes was interpreted as qualitatively more human-like with some understanding of others' mental states, desires, and intentions [39]–[41], the cognitive abilities shown by monkeys were mainly attributed to rapid learning capacities [37], [42]. However, a recent study suggests that not only apes but also monkeys are able to form mental representations [29]. We tested the same olive baboons and long-tailed macaques in a quantity discrimination task with food and non-food items and found that the performance of the monkeys was influenced by their representation of the items as reward or choice stimuli and not by their quality (being edible or not).

Yet, we do not claim that the cognitive abilities of monkeys and apes are generally similar, either. It could also be the case that only the cognitive competencies in the items that were tested in the PCTB do not differ substantially. The PCTB mainly consists of experiments from developmental psychology that were designed to unravel the ontogeny of human specific skills (Social learning, Communication, Theory of Mind). Thus, it is possible that the tasks have been too difficult to allow a measurable difference between monkeys and apes. On the other hand, the good performance in the physical domain may constitute a ceiling effect. In other words, these tasks were structurally simpler and thus yielded high scores in many of the tasks. Furthermore, although the monkeys were able to solve most of the tasks in the physical domain, it is not clear whether they really had an understanding of the underlying physical properties. So it may well be that one would find differences between species when analyzing more specifically how the subjects solved the different tasks [43].

Another issue that needs to be evaluated critically is the fact that in the original study [18], two to three year old children were compared to mainly adult apes and monkeys, respectively. It would be highly desirable to assess the performance of adult humans in these tasks to obtain a more comprehensive picture. Moreover, in contrast to non-human primates, children are tested by members of their own species (for a critical review see e.g [44]). Thus, such species comparisons often cannot control for a number of serious confounds, which should be held in mind.

Despite these limitations, the idea to test different species in such a large battery of tasks is a productive approach in comparative cognition studies. There is a caveat, however. As Tinbergen [45] already pointed out, the same test for a different species may not be the same test. Above all, this applies to situations where a given test yields different results (as in the case of the children vs. the nonhuman primates). In such instances, it is necessary to further investigate why a given species apparently fails in a certain test, and to develop experiments with a high ecological validity for each species. For instance, baboons and macaques hardly ever use sticks to retrieve food, so it is perhaps not surprising that they failed in this task.

A recent paper on comparative phylogenetic methods strongly encourages the integration of comparative psychology and evolutionary biology [19]. It is particularly important to consider variation in the species' socio-ecology in such analyses. Furthermore, future studies should also take care of variations in physiological characteristics between species, as e.g. in visual fields or attention patterns. Differences between species may be influenced by perceptual rather than cognitive differences. In addition, it would be highly desirable to compare the abilities of the same species in different labs and settings, to obtain a comprehensive understanding of the variability between and within species. Although we did not find any significant correlations between the temperament and inhibition control measures respectively and the cognitive performance of the monkeys in our study, taking such additional factors into account has proven to be useful when comparing the cognitive abilities of different species [18], [24].

In conclusion, our study provides the first evidence that the cognitive skills of monkeys and apes are much more similar than expected both in the social and physical domain, at least in the tests of the PCTB. Hence, our results support the view of an accelerated evolution of social respectively cultural intelligence in humans [18]. We could furthermore show that it is essential to use a wide range of experiments when comparing the cognitive capacities of different species. Using only a subset of experiments (e.g. only spatial displacements) would have led to completely different conclusions. Thus, future comparative approaches should also consider including multiple cognitive experiments of different domains.

## Supporting Information

File S1 Detailed description of the experiments used in the PCTB.
(PDF)

File S2 Detailed description of the methods used to assess temperament, inhibitory control and rank.
(PDF)
