# Supplementary material for: Old World Monkeys Compare to Apes in the Primate Cognition Test Battery
Source: PLoS One. 2012 Apr 2;7(4):e32024. doi: 10.1371/journal.pone.0032024 (PMC3317657; doi:10.1371/journal.pone.0032024)
Supplement: File S1 — Detailed description of the experiments used in the PCTB. (PDF) [file pone.0032024.s001.pdf]

File S1

Supporting Information on Methods of the PCTB

Primate Cognition Test Battery

The tasks and procedures were adopted from the study by Herrmann et al. (2007). When we did changes in the experimental procedure these are marked in bold and italic face. Otherwise the methods used were the same (therefore we also used similar wording to describe the tasks in order to avoid confusion) and only the size of the material was adjusted to be operable for the baboons and long-tailed macaques, respectively.

Physical Domain

1. Space

a. Spatial Memory

Three cups were placed in a row on the platform in front of the testing cage. The experimenter then showed the subject two rewards and placed them under two of the three cups in full view of the subject. Then the platform was pushed towards the subject and it was allowed to make up to two choices in succession. If, however, the subject chose the empty cup first, it was not allowed to make further choices. The response was counted as correct when the subject had chosen both baited cups in succession.

b. Object Permanence

Three cups were placed in a row in front of the testing cage. An additional small opaque cup was placed on the far left ***or far right side***, respectively of the platform. The experimenter placed a reward under this small cup while the subject was watching. The small cup was then moved towards one of the larger cups, which was slightly lifted by raising the side not facing the subject. The experimenter then made a swapping movement with the small cup as if swapping the reward under the larger cup. There were three possible displacements performed:

Single displacement: The experimenter moved the small cup hiding the reward under one of the three cups as described above, swapped the reward under it and did not touch the other two cups.

Double adjacent displacement: The experimenter moved the small cup hiding the reward under two adjacent cups in succession as described above, left the reward under one of these cups without touching the third cup.

Double non-adjacent displacement: The experimenter moved the small cup hiding the reward under the left and right cup in succession as described above and left the reward under one of them. The cup standing in the middle was not touched.

Single displacement touch: ***The experimenter moved the small cup hiding the reward under one of the three cups as described above and left the reward under this cup. However, in this condition the experimenter touched the other two cups with her hand to examine whether the subjects only chose***

the last cup touched by the experimenter or really took into account where the smaller cup was moved to.

After moving the small under the specific larger cups the experimenter lifted the small cup to show the monkey that the small cup was now empty. The platform was pushed forward and the monkey was allowed to choose either one cup (in the single displacement item and control condition) or up to two cups (in the double displacement items). However, if the subject chose a cup under which the smaller cup was not moved during the demonstration, no further choices were allowed. A correct response was counted when the monkey had chosen the baited cup before choosing a cup which was not manipulated at all.

c. Rotation

Three cups were placed in a row on a tray, which was then placed on the platform in front of the testing cage. The experimenter showed a reward to the monkey and placed it under one of the three cups while the subject was watching. Then the tray was rotated in three possible ways:

180° middle: The reward was placed under the middle cup, and the tray was rotated 180° in clockwise ***or counterclockwise*** direction, respectively. After the rotation, the reward was located in the same location as it was initially placed.

360°: The reward was placed under either the left or right cup, and the tray was rotated 360° in clockwise ***or counterclockwise*** direction, respectively. After the rotation, the reward was located in the same location as it was initially placed.

180° side: The reward was placed under either the left or right cup, and the tray was rotated 180° in clockwise ***or counterclockwise*** direction, respectively. After the rotation, the reward was located on the opposite side of where it was initially placed.

After the completed rotation the subject was allowed to choose one cup. A correct response was scored when the subject chose the baited cup first.

d. Transposition

Three cups were placed in a row on the platform in front of the monkeys' testing cage. The experimenter showed a reward to the monkey and placed it under one of the three cups while the subject was watching. Then one of three possible manipulations was performed:

Single transposition: The experimenter switched the position of the baited cup with one of the empty cups. The third cup was not touched.

Double unbaited transposition: The experimenter switched the position of the baited cup with one of the empty cups. Then the positions of the two empty cups were switched.

Double baited transposition: The experimenter switched the position of the baited cup with one of the empty cups. Then she switched the position of the baited cup again with one of the empty cups.

After the transpositions were completed the subject was allowed to choose one cup. A correct response was scored if the monkey chose the baited cup first.

2. Quantities

a. Relative Numbers

The experimenter placed two plates on the platform in front of the testing cage and put up an occluder to prevent the monkeys from watching the baiting procedure. Then she baited the plates with different amounts of equal sized food pieces (half a peanut or raisin was used as a unit). The experimenter then placed the plates in the middle on the platform and removed the occluder so the subjects could see the amounts lying on each plate. After ~5 seconds had passed and the subject paid attention, the experimenter moved the plates simultaneously to the sides of the platform, one to the right and one to the left. The sliding table was pushed against the Plexiglas panel and the subject was allowed to choose and received all food pieces lying on the respective plate. Each subject received one trial for each of the following pairs of numbers (***the order was randomized)***:

1:0, 1:2, 1:3, 1:4, 1:5, 2:3, 2:4, 2:5, 2:6, 3:4, 3:5, 3:6, 3:7, 4:6, 4:7, 4:8 and four control conditions 1:1, 2:2, 3:3, 4:4 to monitor any laterality bias, i.e. going on the same side on every trial.

A correct response was scored if the subject chose the larger quantity first.

b. Addition Numbers

The experimenter placed three plates on the platform in front of the testing cage and put up an occluder to prevent the monkeys from watching the baiting procedure. Then she baited the three plates with different amounts of reward (same as in Relative Numbers) covered them with lids, and placed them in the middle of the platform. After the occluder was removed, the experimenter lifted the lids of the two outer plates simultaneously. After ~5 seconds had passed, the experimenter covered the two outer plates again and uncovered the plate in the middle. The monkeys were able to view the amount lying on the middle plate for ~5 seconds. Then the experimenter transferred the rewards from the middle plate to one of the side plates. During the transfer the subject could not see the content of the side plates. Then the experimenter removed the empty plate in the middle and the subject was allowed to choose between the two covered plates on the outer sides. Each subject received ***two*** trials for each of the following pairs (***the order was randomized***):

1:0 + 3:0 = 4:0; 6:1 + 0:2 = 6:3, 2:1 + 2:0 = 4:1, 4:3 + 2:0 = 6:3, 4:0 + 0:1 = 4:1, 2:1 + 0:2 = 2:3, 4:3 + 0:2 = 4:5. ***Each of the combinations was presented with the resulting higher number being once on the left and once on the right side, resulting in 14 trials in total.***

A correct response was scored if the subject chose the larger quantity first.

3. Causality

a. Noise

The experimenter placed two cups on the platform in front of the testing cage and put up an occluder to prevent the monkeys from watching the baiting procedure. Then she put a reward (peanut) in one of the two cups and closed them with a lid. After the occluder was removed one of two possible manipulations were performed:

Noise full: The experimenter shook the baited cup three times so that the food rattled inside and only lifted the empty cup without shaking it. **Starting with the baited or empty cup was randomized.**

Noise empty: The experimenter shook the empty cup (producing no sound) three times and then lifted the baited cup without shaking it. **Starting with the baited or empty cup was randomized.**
After the manipulations the subject was allowed to choose one cup. A correct response was scored if the subject chose the baited cup first.

b. Shape

The experimenter put up an occluder and placed two identical pieces of cardboard or cloth, respectively on the platform in front of the subjects test cage. Then the experimenter placed a reward (peanut) underneath one of the two identical objects causing a visible inclination or bump, respectively. After that the occluder was removed, and the subject was allowed to make one choice.
Board: The experimenter hid the reward underneath one of two cardboard boards (15 x 12 cm). The reward caused a visually apparent inclination as it was placed on the food (the other board remained flat on the table).
Cloth: The experimenter hid the reward underneath one of the two pieces of cloth (15 x 10 cm). The reward made a visible bump under this piece of cloth (the other cloth remained flat on the table).
A correct response was scored if subjects chose the baited board or cloth first.

c. Tool Use

A reward was placed on a table approximately 20 cm out of reach of the subject in front of their cage A wooden stick (20 cm in length) was provided for the subject. In this experiment no plastic panel was used. The animals could handle the stick through the wire mesh of their cage. To be successful the subject had to use the tool to retrieve the out of reach object or food within two to three minutes.
A correct response was scored if the subject was able to retrieve the reward.

d. Tool Properties

In these experiments a plastic panel with two oval openings on the left and right side was attached to the Plexiglas panel. The experimenter put up an occluder and placed two different tools on the platform in front of the testing cage. One tool was functional and could be used to retrieve a reward associated with it (e.g. lying on top of it), whereas the second tool was non-functional and could not be used to obtain the associated reward. In total five different objects were used:
Side: The experimenter put two identical pieces of cloth (15 cm x10 cm) on the platform behind an occluder and placed a reward on top of one cloth piece, whereas the other reward was placed directly next to the other cloth piece (i.e. making the second tool ineffective for retrieving the food). After the occluder was removed, the subject could only retrieve the reward by pulling the piece of cloth with the reward on top of it.
Bridge: The experimenter put two identical small plastic bridges over each of the far ends of the two identical cloth pieces behind an occluder. One reward was then placed on top of the bridge (making the tool ineffective in retrieving the food), the other reward was placed on the cloth underneath the bridge. After removing the occluder, the subject could only obtain the reward by pulling the cloth with the reward placed directly on it.

Ripped: The experimenter put up an occluder and placed a rectangular, intact cloth piece (15 cm x 10 cm) on one side of the table, and two smaller cloth pieces (9.5 cm x 10 cm and 4.5 cm x 10 cm) on the other side, arranging the small pieces of cloth in a way that there was a 1 cm gap between them. Then one reward was placed on top of the far end of the intact cloth, and the other reward was placed on the out of reach piece of the two disconnected pieces (making the tool ineffective to retrieve the reward). After removing the occluder, the subject could only acquire a reward by pulling the large, intact cloth piece.

Broken wool: The experimenter put up an occluder and placed two strings of wool on the platform, from which one was cut into two pieces. Like in the Ripped cloth condition both strings were arranged in a way that the gap was visible, but that both resulted in an equal length. A peanut was tied to the far end of the wool strings out of the subject's reach. After removing the occluder, the reward could only be retrieved by pulling the intact piece of wool.

Tray circle: The experimenter placed two small cardboard trays (6 cm x 6.5 cm) on the platform behind an occluder. One tray had a hole cut out of it that formed a circle (3.5 cm in diameter) the other tray had a u-shaped hole, thus open to the end facing away from the monkey. A string was attached to both trays that could be used to pull the tray and the reward within reach. Then a reward was placed into the holes of each tray. In case of the u-shaped hole the tray surrounded the food but did not hold it. After removing the occluder, the subjects could only obtain the reward if they pulled the rope which was attached to the tray with the circle-shaped hole in it.

A correct response was scored if the subject first chose the functional tool by pulling it.

Social Domain

1. Social Learning

Before we conducted the social learning tasks, we established a baseline with 3 olive baboons and 3 long-tailed macaques. None of the six subjects solved the problem with the same means we demonstrated in the following three tasks (i.e. making it likely that any reproduction of the demonstrations described below are due to social influences). In the test conditions the subjects were given two minutes to solve the problem after the experimenter demonstrated the solution. To count as a correct response the subject had not only to obtain the reward but do so by using a highly similar procedure as the one demonstrated by the experimenter.

a. Paper Tube

The experimenter placed a reward inside a 30 cm long transparent plastic tub, which was coverd by two pieces of paper attached over both ends. Then she stood in front of the monkeys' cage and demonstrated how to open the tube: She held the tube in one hand and poked a hole into the paper with a finger. Then she ripped the paper further by twisting her finger in the tube. After that she tilted the tube and let the reward fall in her hand. After the demonstration she handed an identical tube to the subject.

b. Banana Tube

A slice of banana was placed in the center of a 30 cm long transparent Plexiglas tube. The banana was trapped in the tube and could only be retrieved by applying a specific force. The experimenter stood in front of the monkeys' cage and showed them how to retrieve the banana by banging one end of the Plexiglas tube on the floor. After the successful demonstration, she handed an identical tube with a banana inside to the subject.

c. Stick Tube

A 15 cm long opaque plastic tube with caps on each end was baited with a reward (peanut). One of the caps had a small hole in it but was tightly attached to the tube, whereas the other cap had no hole but could be removed. The experimenter stood in front of the monkeys' cage and demonstrated how to open the tube: She inserted a wooden stick through the cap with a hole, and pushed the stick through the hole which forced the cap on the other end to fall off. After the successful demonstration she handed an identical grey tube to the subject.

2. Communication

a. Comprehension

The experimenter placed two cups on the testing platform behind an occluder, one on the left and the other on the right side. Then she hid a reward under one of the cups. After removing the occluder she gave one of three social cues:

Look: The experimenter sat behind the platform and alternated her gaze between the subject and the baited cup three times while calling the subject's name. After these gaze alternations she continuously looked towards the cup until the subject chose.

Point: The experimenter sat behind the platform and continuously pointed to the baited cup with the extended index finger of her cross-lateral hand. At the beginning of the point, she alternated her gaze between the subject and the cup three times while calling the subject's name and then only stared in the baited cup's direction.

Marker: The experimenter held an iconic photo marker, which depicted the reward, in her hand and alternated her gaze three times between the photo and the subject while calling the subject's name. Then she placed the photo on top of the baited cup.

After the cue the subject was allowed to choose one cup. A correct response was scored if the subject chose the baited cup first.

b. Production: Pointing Cups

In the following task two experimenters were needed (E1 & E2). Two cups served as hiding places for a food reward. These cups were placed on the outer part of the platform in front of the testing cage. The hiding places were spread apart (ca. 50cm (macaques), ca. 70cm (baboons)) and both equidistant from the subjects' starting point between the two hiding places. The second experimenter (E2) entered the testing area, placed a reward under one of the two cups while the subject was watching, and then left the area. Then E1 entered the testing area and centered the monkey by giving her a piece of food between the two cups through the middle hole in the Plexiglas panel. Then E1 stood equidistant to

both cups and waited until the subject approached one cup and pointed towards it through a hole in the Plexiglas panel. A correct response was scored if the subject chose the correct cup within one minute.

c. Production: Attentional State

In the following task again two experimenters were needed (E1 & E2). The second experimenter (E2) entered the testing area and placed a reward out of reach but in front of the subjects' cage on its right or left side. Then E2 left the area and E1 entered, but stood on the end of the room opposite of the reward and thus did not notice the reward on the floor. E1 stood and looked in four different ways:

Away: E1 turned around and looked away from the reward. When the monkey approached E1 from her front in order to see each other within 20 seconds (20s), E1 turned around and waited for the subject to direct her attention to the reward. If the subject went back to the reward's location and indicated the reward within 20s, E1 handed the reward to the subject.

Towards: E1 looked towards the reward. When the monkey approached the reward and directed E1 attention towards the reward within 20s, E1 handed it over to the subject.

Away Body-facing: Identical to Away, except that E1's body faced toward the reward and only the face was turned away. When the monkey approached E1 and directed her attention towards the reward within 20s, E1 handed it over to the subject.

Towards Body-away: Identical to Towards, except that E1's body was turned away and only the face was directed towards the reward. When the monkey approached the reward and directed E1 attention towards the reward within 20s, E1 handed it over to the subject.

3. Theory of Mind

a. Gaze Following

The experimenter sat in front of the subject and gave it a piece of food to attract its attention. When the monkey sat and looked at the experimenter, she started the trial. The gaze cue was conducted in three different ways: (***which were conducted on a different day within the test battery to minimize any kind of habituation***)*:*

Head + Eyes: The experimenter called the subject's name and showed them a piece of food. Then she hid the food in her hand, which remained in front of her body. She then looked up with both her head and eyes for ~10s.

Back: The experimenter sat with her back facing the subject. She called the subject's name and showed them a piece of food. Then she hid the food in her hand, which remained in front of her body. She then looked up to the ceiling for ~10s. Within the ~10s she looked back over her shoulder at the subject three times to ensure that the subject was still paying attention. If the subject was not paying attention when the experimenter looked the second time, the trial was repeated.

Eyes: The experimenter called the subject's name and showed them a piece of food. Then she hid the food in her hand, which remained in front of her body. She then glanced up at the ceiling for ~10s while her face was still facing the subject.

A correct response was obtained if the subject followed the gaze of the experimenter.

Straight: To control whether the subjects also gaze upwards without the experimenter looking up, we conducted this control condition. The experimenter called the subject's name (and showed them a piece of food before hiding it in her hand, which remained in front of her body). However, instead of gazing upwards the experimenter looked straight forward at the subjects' chest.

b. Intentions

In these tasks two experimenters were needed (E1 & E2). E1 put up an occluder and placed two cups on the platform in front of the testing cage. Then she hid a reward in one of the two cups. After removing the occluder, E2 manipulated the cups in one of two ways:

Trying: E2 reached for the baited cup and tried in vain to remove the lid while looking at the cup.

Reaching: A Plexiglas barrier blocked E2's access to the cups. Therefore, E2 unsuccessfully tried to reach the baited cup by extending the equilateral arm, looking at the correct cup. She continued to give this cue until the subject indicated a choice.

After each demonstration E1 approached the table after ~3s and pushed the platform forward so that the subject was allowed to make a choice. To count as a correct response subjects had to choose the baited cup first.

Table S1: Number of trials for each task and item

**Task/Item**	**Trials**
**Spatial memory**	**6**
**Object Permanence**	**18**
Single displacement	6
Double adjacent displacement	6
Double non-adjacent displacement	6
**Rotation**	**18**
180° middle	6
360°	6
180° side	6
**Transposition**	**18**
Single transposition	6
Double unbaited transposition	6
Double baited transposition	6
**Relative Numbers**	**16**
**Addition Numbers**	**14**
**Noise**	**12**
Noise full	6
Noise empty	6
**Shape**	**12**
Board	6
Cloth	6
**Tool use**	**1**
**Tool properties**	**30**
Side	6

**Task/Item**	**Trials**
**Social learning**	**3**
Paper tube	1
Banana tube	1
Stick tube	1
**Comprehension**	**18**
Look	6
Point	6
Marker	6
**Pointing Cups**	**8**
**Attentional State**	**4**
Away	1
Towards	1
Away Body-facing	1
Towards Body-away	1
**Gaze following**	**9**
Head & Eyes	3
Back	3
Eyes	3
**Intentions**	**12**
Trying	6
Reaching	6

Bridge	6
Ripped	6
Broken wool	6
Tray circle	6
