# Supplementary material for: Old World Monkeys Compare to Apes in the Primate Cognition Test Battery
Source: PLoS One. 2012 Apr 2;7(4):e32024. doi: 10.1371/journal.pone.0032024 (PMC3317657; doi:10.1371/journal.pone.0032024)
Supplement: File S2 — Detailed description of the methods used to assess temperament, inhibitory control and rank. (PDF) [file pone.0032024.s002.pdf]

File S2

Assessment of Temperament, Inhibitory Control and Rank

Temperament test

To assess differences in temperament between the monkeys we also used the same methods as Herrmann et al. (2007). To assess differences in the temperament of the subjects we tested their reaction to novel objects, persons and foods. When appropriate we also used the same wording to describe the tasks as Herrmann et al. The test situation varied concerning 1) the nature of the different items shown (humans, objects or food pieces), 2) whether the items were presented alone or in combination (e.g. human moving a novel object) and 3) whether the objects were moved or not or could be touched during their presentation (e.g. item was moved from left to right by the experimenter, etc.). We measured whether the subjects approached the new items, how fast they did that and whether they tried to touch the presented objects.

Procedure and Design

Similar to the temperament tests done by Herrmann et al. (2007) each monkey participated in 29 different items which could be grouped into four categories: human, object, food/reward and non-human (see Table S2). For the presentation of each item the unfamiliar experimenter (E1) sat in front of the monkeys' cage (excluding the familiar human and non-human condition). A second, familiar experimenter (E2) made sure that the subject was at a designated starting point by offering food. When the monkey was in the correct place E1 presented the different stimuli for 30 seconds each. The subjects received one session per day and the objects were presented in the same order across all subjects. On the first day (**Visible**) the subjects were only able to view the experimenter sitting behind the table and the different items were placed on the table. On this day we also conducted two additional non-social trials in which the monkeys could either view the table alone or when a bright red spot was placed on the table top before E1 left the area. On the second day (**Movement**) the different items were moved from left to right during the 30s of presentation by the experimenter. On the third day (**Touch**) the items were put close to the monkeys' cage so that the subjects were able to touch them.

During all of the experiments a camera filmed a predetermined 'visible area' (including the items and about 2sqm of the cage near the items). From the videos we then recorded three measures: latency (time to come into the proximity of the object), duration (time spent near the object), and proximity (how close the monkeys approach the object).

Table S2: Items and Methods used in the Temperament Test.

	**Category**	**Item**	**Description**
**Visible**	Human	- Familiar<br>- Non-Familiar (E1)	The Person sat behind the table facing the mesh.
	Object	- Orange ashtray<br>- Plastic Beetle	E1 sat behind the table, hands on her lap with the object placed in the middle of the table.

		- Police car	In the police car condition E1 held the remote control and pressed the horn button ten times.
	Food	- Undesirable food<br>- Fruit piece<br>- 3 Peanuts<br>- Lemon	E1 sat behind the table, hands on her lap with the food placed in the middle of the table.
	Non-Human	- Red spot<br><br>- Nothing	E1 placed a red spot in the middle of the table and left.<br>Nothing is on the table and E1 is out of sight.
**Movement**	Human	- Hand<br>- Body	E1 sat behind the table, moved her right hand from the left side to the right side.<br>In the body condition E1 nodded up and down.
	Object	- Orange Ashtray<br>- Plastic Beetle<br>- Police car	E1 sat behind the table, moved the object from the left side to right side and back on the table.<br>In the police car condition E1 let the car drive to the on the table.
	Food	- Undesirable food<br>- Fruit piece<br>- 3 Peanuts<br>- Lemon	E1 sat behind the table, moved the food from the left side to right side and back on the table.
**Touch**	Human	- Hand	E1 sat behind the table, put her right hand as a fist on the table.
	Object	- Orange Ashtray<br>- Plastic Beetle<br>- Police car<br>- Box	E1 sat behind the table, hands on her lap with the object placed on the table within reach of the subject.
	Food	- Undesirable food<br>- Fruit piece<br>- 3 Peanuts<br>- Lemon	E1 sat behind the table, hands on her lap with the food placed on the table within reach of the subject.

Analyses and Results

To analyse the differences between baboons and macaques in the three measures (i.e. latency, duration or proximity) we used the same methods as for the performance in the different PCTB tasks (MANOVA and ANCOVA). To compare the results of these temperament tests to the performance in the PCTB we first determined the most indicative temperament variable. Therefore, we did a variance component analysis to examine which factors (i.e. individual, item, or procedure) best explained the variation in the three measurements and compared this to the performance in the PCTB (Pearson correlations).

The variance component analyses revealed that the measure “Duration” accounted for the largest differences between individuals. With this measure we then conducted Pearson correlation analyses with the performance in the physical and social domain, which were all not significant.

Concerning species there was a significant difference between the baboons and macaques (MANOVA with species and sex as between-subject factor and scores in the three measurements as dependent variables; F (3, 11) = 28.72, p < .001, η^2= .887). Post hoc tests showed that the baboons spent more time next to new objects than the macaques (p < .001) and approached new stimuli faster (p = .002). There was also a significant effect of sex (Wilk’s Lambda, F (3, 11) = 5.89, p < .012, η^2=

.617) and interaction between species and sex (Wilk's Lambda, F (3, 11) = 5.05, $p < .019$, $\eta^2 = .579$) with male baboons staying significantly longer near new items than female baboons (Posthoc-test, p = .011) and longer than male and female macaques (Posthoc-tests, $p < .001$). Furthermore, female macaques took significantly longer to approach a new item than female (Posthoc-test, p = .023) and male baboons (Posthoc-test, p = .002). When controlling for age these differences still remain significant (ANCOVA with species and sex as between-subject factor, age as covariate and scores in the three measures as dependent variables, all $p < .034$).

Inhibitory control test

To examine whether the amount of inhibitory control correlates with the monkeys' performance in the PCTB, we conducted the same inhibitory control tasks as Herrmann et al. (2007). We conducted three additional trials within the spatial memory tasks. Rewards were placed under two out of three cups while the subject was watching. For these trials, however, only the two outer cups were baited, while the middle cup was left empty. If the subject first chose one of the outer cups it was allowed to make a second choice. If, however, it chose the middle cup first, no further choices were possible. A correct response was scored when the monkey chose the two outer cups in succession while skipping the middle cup.

We found no significant difference between the performance of the baboons and macaques (Mann-Whitney U-Test, z = 0, p = 1).

Rank

To examine whether rank has an influence on the subjects' performance we completed focal observations for each individual of the long-tailed macaques, which allowed us to classify them as high, middle or low ranking (see Table S3). Focal observations lasted 120 min (6 x 20 min) for each animal. Rank was calculated by subtracting the number of events in which aggression was received from the number of events aggression was given, corrected for the total number of aggressive events observed per animal. The resulting score determined whether a subject was considered as high, middle or low ranking. The rank of the baboons was estimated by personal observations (VS) over 2.5 years.

Table S3: Results of the focal observations done on the monkeys and their classification as high, middle or low ranking.

**Subject Macaque**	**(Aggression given-Aggression received)/N of Aggression**	**Rank**
Su	0.92	h
Ma	0.76	h
Is	0.70	h

**Subject Baboon**	**Rank**
Pk	h
Ms	h
Bh	m

Po	0.26	m
Pa	0.11	m
Sa	0.11	m
Pi	0.00	m
Se	-0.08	m
Le	-0.11	m
So	-0.54	l
Sam	-0.57	l
Li	-0.78	l
Sun	-1.00	l

- > 0.5 = High
- -0.5 – 0.5 = Middle
- < -0.5 = Low

Jg	m
Tg	l
